# Large herbivores and abiotic drivers jointly shape spatiotemporal grassland dynamics in a subalpine ecosystem

**DOI:** 10.1038/s41598-026-45843-0

**Published:** 2026-04-14

**Authors:** Daniel Oro, Aleix Ferrer-Vilaplana, Joan Bauzà, Meritxell Genovart, Moisès Guardiola, Giulio Tirabassi, Arantza Aldezabal

**Affiliations:** 1 Department of Ecology and Complexity, CEAB (CSIC), Accés Cala Sant Francesc 14, Blanes, 17300 Catalonia Spain; 2 Servei de Sistemes d’Informació Geogràfica i Teledetecció, Universitat de les Illes Balears, Palma, Spain; 3 Unit of Botany, Department of Animal and Plant Biology and Ecology, Universitat Autònoma de Barcelona, Bellaterra, Spain; 4 Department of Computer Science, Applied Mathematics and Statistics, Universitat de Girona, Girona, 17003 Spain; 5 Landare Biologia eta Ekologia Saila, Zientzia eta Teknologia Fakultatea, Euskal Herriko Unibertsitatea (UPV-EHU), 644 p.k, Bilbo (Bizkaia), Euskal Herria, 48080 Spain

**Keywords:** Extensive grazing, Climate-vegetation interactions, Vegetation indices (SAVI), Herbivore foraging, Non-linear response, Seasonal dynamics, Subalpine grasslands, Ecology, Ecology, Environmental sciences

## Abstract

Managed grazing has a larger geographic extent than any other global form of land use. In subalpine grasslands, the spatiotemporal dynamics of vegetation during its growing season may depend on grazing intensity, the type of vegetation and abiotic drivers, as well as their complex interactions. Here, we examine how free-range grazing cows and abiotic factors (climate and topography) drive grassland dynamics over three growing seasons in a Pyrenean valley. We combine high spatial resolution satellite imagery to estimate our response variable (the Soil-Adjusted Vegetation Index (SAVI), a proxy of vegetation greenness and biomass that correlates with forage quantity and quality), with GPS-tracking of cows (80–85 cows per year), the primary herbivores in the valley. We generated a detailed vegetation map and used GPS accelerometer data to separate grazing from resting cow activities, the latter resulting in higher trampling and nutrient deposition on vegetation. Results indicate that cows showed a clumped spatial distribution, consistent with collective behaviour and a heterogeneous use of the available space. SAVI showed clear seasonal cycles, peaking in early summer and declining to winter dormancy. Grassland dynamics were influenced by climate variability, both seasonal (solar radiation and temperature) and non-seasonal (soil water content, precipitation, and wind). The other drivers were also selected in the best multiple regression model, which explained 46% of SAVI temporal variance. Both cow activities had negative impacts on vegetation, with lower SAVI values associated with resting; grazing showed non-linear negative effects on SAVI, varying synergistically with elevation, slope, and orientation. The nonlinearity resulted from a steep initial decline in SAVI with grazing, followed by a diminishing negative effect as grazing intensity increased. These findings reveal the complex interplay between biotic and abiotic drivers in SAVI dynamics, emphasising the role of large herbivores in the ecological processes in subalpine ecosystems under varying environmental conditions.

**Supplementary Information:**

The online version contains supplementary material available at 10.1038/s41598-026-45843-0.

## Introduction

The dynamics of plant communities are driven by several ecological and demographic processes (e.g., density dependence, facilitation, dispersal) and by their interactions with the environment, both abiotic and biotic^1,2^. In temperate and mountain regions, seasonal climate constrains growing length and productivity, while topography and edaphic conditions modulate plant growth and community composition and may generate strong environmental gradients over small spatial scales^3^. Alongside these abiotic controls, herbivores exert top-down control on plant communities and play an important role in their ecological dynamics^4,5^. In terrestrial ecosystems, large herbivores have a particularly strong capacity to shape vegetation structure through defoliation, defecation, trampling, and biogeochemical cycling, and to hamper tree and bush encroachment^6^. In this context, managed grasslands cover a large fraction of the terrestrial surface and are among the most widespread forms of land use worldwide^7^. Thus, livestock management may exert significant ecological effects on ecosystems, including inhibiting or, under some management regimes, facilitating woody encroachment, as well as influencing processes such as woody encroachment, deforestation, eutrophication, and desertification, depending on grazing intensity and on edaphic, climatic and biogeographic context^7–10^. At the plant-community scale, grazing affects vegetation through a combination of biomass removal, mechanical disturbance by trampling, and fertilisation via dung and urine inputs. Nutrient inputs can also be highly spatially aggregated when herbivores repeatedly defecate in the same patches, creating persistent biogeochemical spatial heterogeneity as documented in cattle systems^11–13^. Importantly, the relative importance of these pathways may differ depending on whether animals are grazing or resting, so that consumptive and non-consumptive effects of herbivory can produce contrasting vegetation responses in grasslands.

To study the effects of large herbivores on grassland dynamics, the commonest approach has been the experimental exclusion of livestock to compare ecological communities between grazed and ungrazed patches^14–19^. However, such studies generally assume that replicated grazed plots experience similar grazing pressure. High-resolution movement studies using advanced tracking technologies reveal that livestock grazing and resting exhibit strong spatiotemporal heterogeneity, driven by seasonality, micro-scale vegetation patch characteristics and species composition, topographic features of the terrain, and the spatial configuration of resources (e.g. grass, water, shelter)^20–22^. These movement patterns, far from being random, generate patchiness of intense herbivory and trampling around recursive resting sites and resource patches, while leaving other areas lightly used^23–25^. Additionally, high-resolution tracking allows for the incorporation of the social behaviour of livestock, which may increase spatial heterogeneity in grassland dynamics. Consequently, heterogeneous and collective space use may result in spatial mosaics of vegetation productivity and recovery, with patches under recursive pressure showing reduced greenness because repeated trampling and intensive use depress vegetation height and cover and expose more soil, whereas lightly or moderately grazed areas can exhibit vigorous green regrowth compared with ungrazed patches dominated by standing dead vegetation. Despite this, many studies still lack fine-grained spatiotemporal information on where animals move, which vegetation types they use, and what they are doing (e.g. grazing versus resting) at each patch and time. A complementary approach to study grassland spatiotemporal dynamics is remote sensing, which has been widely used to characterise vegetation patterns and dynamics by providing repeated, spatially explicit observations of vegetation state across large and heterogeneous landscapes^26–28^. However, it has only rarely been combined with high-resolution animal movement data, limiting our ability to directly link fine-scale herbivore space use and behaviour (e.g. spatial social clumping) with remotely sensed vegetation dynamics. Spectral vegetation indices such as the Normalised Difference Vegetation Index (NDVI) and the Soil-Adjusted Vegetation Index (SAVI) are commonly used indicators of vegetation greenness and photosynthetic activity, and SAVI in particular reduces sensitivity to soil background effects. Because index values vary with biomass, vegetation structure (e.g. herbaceous vs. shrubby, deciduous vs. evergreen), and the phenology of a given vegetation type, which varies through time (seasonally) and across space (e.g. along elevation gradients), they represent suitable proxies for spatiotemporal vegetation dynamics in grasslands. Previous research has shown that grazing pressure is reflected in vegetation spectral indices, with heavy grazing reducing NDVI or SAVI values due to loss of biomass and greenness, while light or moderate grazing may sustain higher values by promoting compensatory growth^15^. These relationships are often non-linear^29,30^, with thresholds beyond which vegetation cannot recover quickly, leading to sharp declines in spectral indices^30,31^. Furthermore, SAVI values in resting sites where trampling is intense are expected to be strongly reduced.

Here, we study how extensively managed free-ranging cows influence spatiotemporal dynamics of grassland vegetation communities in a subalpine ecosystem in the Pyrenees using two combined high-resolution approaches (satellite images for SAVI indexes and tracking herbivores) together with a detailed vegetation map of the study area that allows SAVI dynamics to be interpreted in relation to vegetation types. We explore three broad questions: first, how do climatic drivers, both variables with strong seasonal cycles (e.g. temperature and solar radiation) and variables showing more irregular temporal variability (e.g. rainfall, wind or soil moisture), explain spatiotemporal SAVI dynamics, and how strong is their influence relative to grazing; second, to what extent does topography (e.g. slope, orientation, elevation) modulate the effects of grazing and climate on SAVI; and finally, how herbivore activity and movement influence spatiotemporal SAVI dynamics and whether this relationship is non-linear. We were particularly interested in potential differences between grazing‑ and resting‑dominated areas, where stronger negative effects were expected.

## Methods

### Study area and herbivore model

**Fig. 1:**
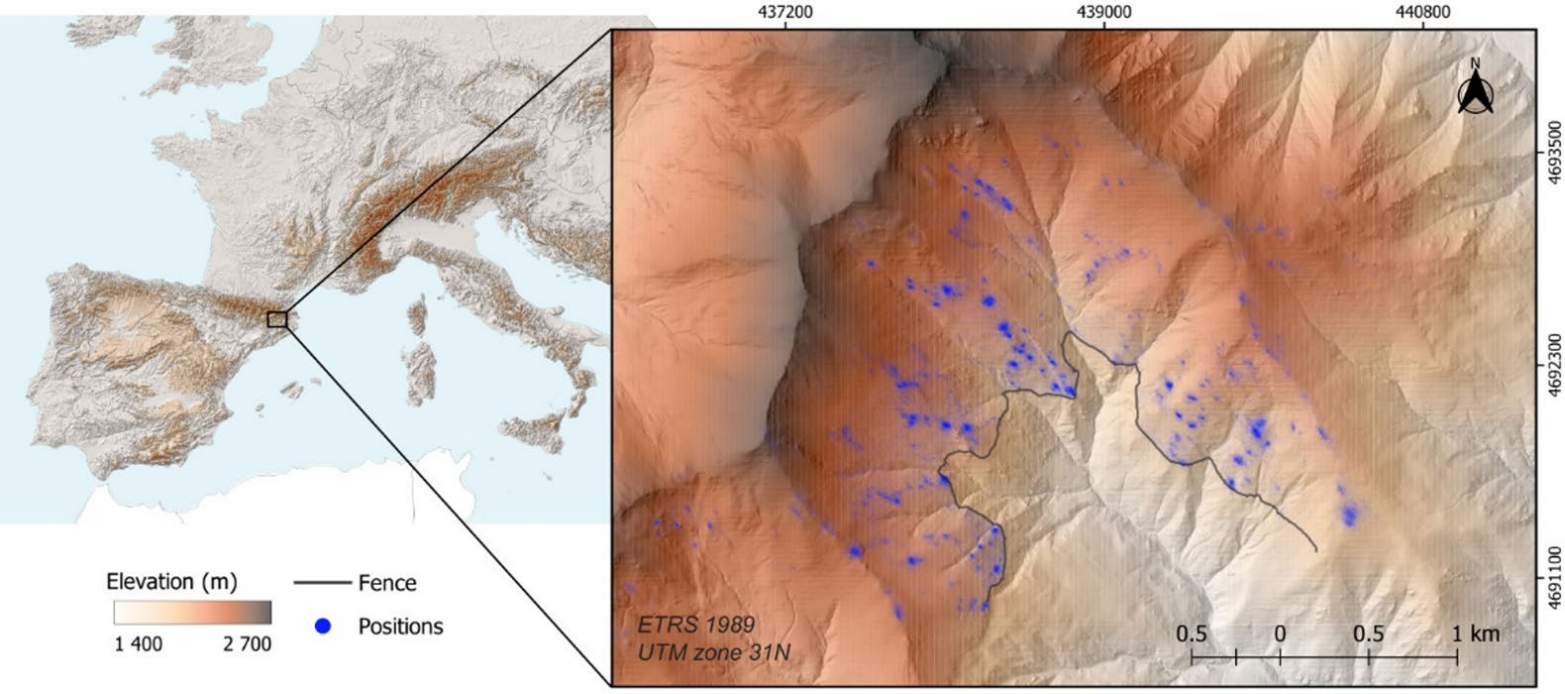
Location of Vall del Catllar in the eastern Pyrenees. The GPS positions of the tracked cows from 2023 are displayed in the upper part of the valley, above the electric fence (refer to the text for details). Cows occupy this part over the summer and autumn seasons. The map also shows the elevation raster and the hydrographic network of the valley. Note that all mapped study pixels are between the fence and the upper ridges of the valley. Thus, no structural barriers prevented cow movement within the valley, although some individuals may occasionally leave the valley through natural passes in the surrounding ridges. Projected coordinate system: ETRS89/UTM zone 31 N (EPSG:25831).

The study was conducted in the hydrographic basin of the Vall del Catllar (42°22’55"N, 2°15’19"E), Eastern Pyrenees (Iberian Peninsula) (Fig. 1). It has a surface area of ​​1275 ha and a perimeter of 15.3 km. Four main water springs emerge in the upper reaches of the valley, giving rise to small headwater streams that converge downstream to form the main river, the Riera del Catllar. Since the study area is under a Mediterranean climate regime, the water flow of the hydrographic basin varies seasonally and inter-annually, typically ranging from 0.1 to 0.32 m^3^s^− 1^^32^. Mean annual rainfall in the valley is relatively high for the southern face of the Pyrenees: 1148 mm (median: 1021 mm; range 896–1579, data from 2018 to 2023 retrieved from a meteorological station in the valley, see below). The area predominantly ranges from elevations between 1400 m and peaks nearing 2700 m a.s.l. (Fig. 1). Most slope ranges are steep and vary between 20º and 30º (range: 10º- 60º) (Supplementary Fig. S1). Vegetation is mainly dominated by grassland communities (Supplementary Fig. S2). To optimise pasture use, cows are extensively managed by moving them to the upper half of the valley in early July each year, and a livestock fence (single-strand electric wire) prevents cows from moving to the lower half until late autumn (Fig. 1).

The study herd holds ca. 220 cows and 4–6 bulls corresponding to the Pyrenean brown cows breed. Together with a small harem of mares (6–7), this is the only cow herd grazing in the valley, which is private property. Between 120 and 140 cows carry calves born at the end of the previous year. Herd size is set to accommodate a heuristic optimisation of the available resources (mainly grass, but also water). Ca. 95 chamois (*Rupicapra rupicapra*) graze in the upper parts and the ridges^33^, although this number may fluctuate daily and seasonally due to the high mobility of these wild herbivores^34^. Moreover, the seasonal presence of livestock in grasslands causes spatial disturbance to chamois, forcing them to move to suboptimal feeding zones^35^.

### Study workflow and data integration

**Fig. 2:**
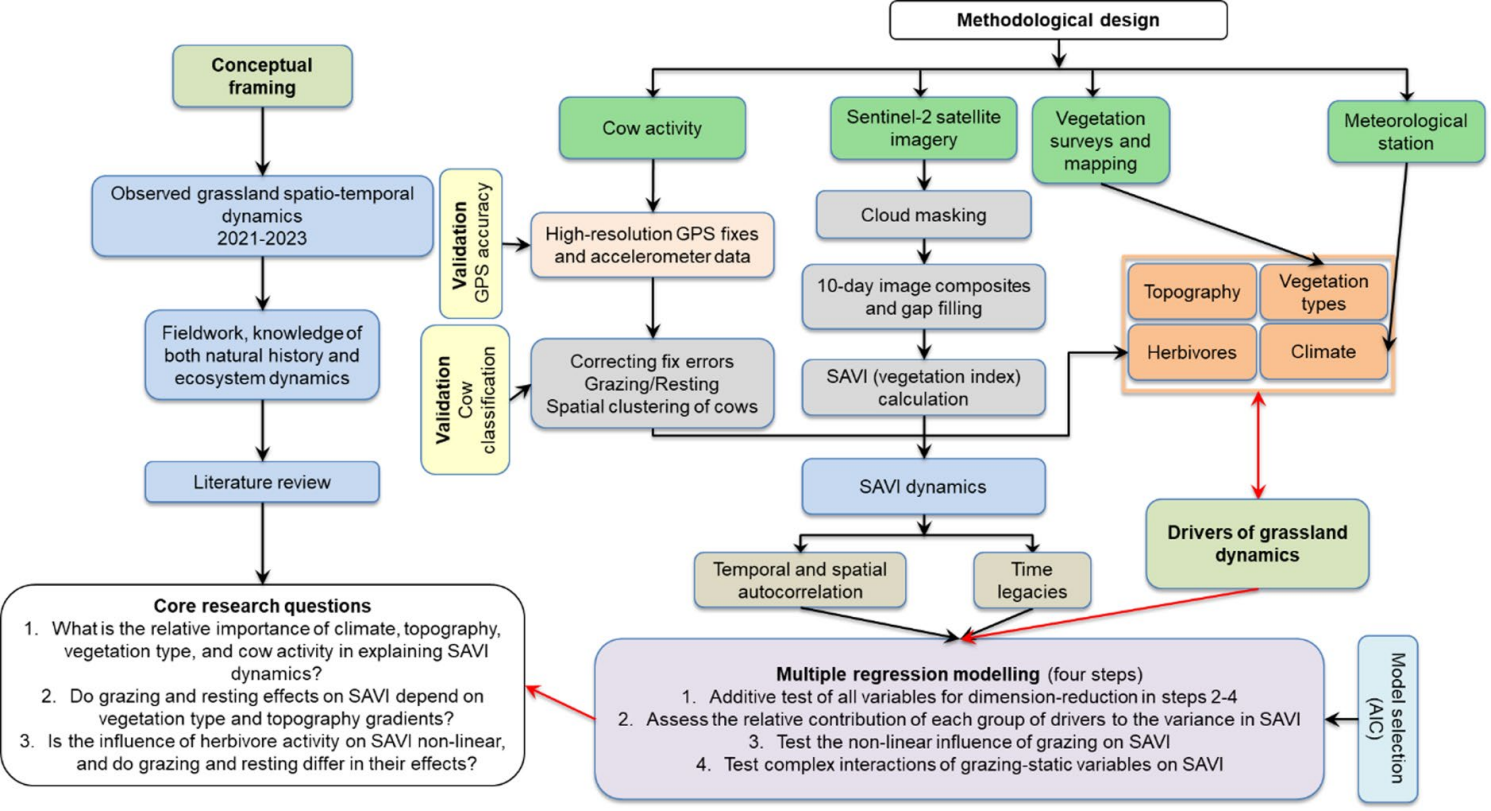
Schematic representation of the workflow data sources, processing steps, validation, and modelling for our integrative research design to study the spatiotemporal dynamics of grasslands in the study area. Red arrows point to the core parts of the study.

Figure 2 shows an overview of the study design, data streams, processing steps, and analytical workflow (Fig. 2), which summarises how cow activity, satellite imagery, vegetation mapping, topography, and climate data were integrated to analyse spatiotemporal SAVI dynamics in the study valley. This schematic overview also illustrates how the different data sources were combined prior to the statistical analyses described below.

### Tracking procedures

To characterise cow space use and activity, we deployed GPS devices (Digitanimal IP67) to 80, 80, and 85 cows in 2021, 2022, and 2023, respectively, during the late spring and early summer seasons, coinciding with the arrival of the cattle herd in the study valley. Devices send fixes every 12 min, which is the maximum temporal resolution to ensure that the battery will last the whole season (from May to late November). We confirmed that the effective GPS fix interval was small and very similar among the study years (mean and standard deviation for 2021, 2022 and 2023: 13.06 ± 2.20 min, 13.89 ± 3.65 min, and 15.36 ± 5.67 min, respectively). Thus, collar performance was stable across years and did not bias interannual comparisons of spatial use metrics. At the end of each season, devices were removed to recharge the batteries. For logistic constraints, most cows were tracked in only one season (64% of the total), whereas a few were tracked in two and three seasons (28% and 8% of the total, respectively).

To evaluate representativeness of tracked individuals, we tested whether tracked cows reflected herd composition regarding having a calf (Log-Linear Model with Poisson regression and log link for the interaction between tracked and calf with year as a factor: Deviance = 2.850, df = 5, *P* = 0.091) and age distribution (same type of model with ages grouped into broader categories with three age classes: 2–3 years old, from 4 to 12 y and older ages: Deviance = 0.13, df = 6, *P* = 0.936). Furthermore, we performed five censuses in July 2023 at early daily hours (when cows are mostly grazing) in which we counted the groups formed in the valley as well as solitary individuals. To define a group of cows, we used 30 times the body length of a cow (ca. 60 m) as the maximum distance for cows to be considered part of the same group. This means that any cow within this distance from another was counted in the same group, regardless of how loosely spaced the cows were. This criterion ensured that even loosely connected cows were grouped based on typical grazing behaviour. To ensure sufficient statistical power, we grouped all the counts into three categories of group size (from 5 to 15 individuals, from 16 to 25, and from 26 to 35) and considered a single category for less social cows (cows on their own and together with a maximum of 4 cows). For each group size, we recorded the number of cows equipped with a GPS device and those without one. GPS-tracked cows were similarly distributed in all groups ($\:{\chi\:}_{3}^{2}$ = 2.320, *P* = 0.509), supporting that fixes from tracked cows were representative of the whole herd and its spatiotemporal distribution.

GPS provided geographic coordinates (latitude and longitude), a timestamp (date-time), and accelerometer data obtained from a three-axis accelerometer: mean positions of each accelerometer axis, standard deviations of each axis, and maximum values of each axis. To ensure GPS data reliability, we initially filtered and removed positions recorded showing speeds < 0 or > 40 km/h between consecutive positions and duplicated fixes. Finally, we quantified positional accuracy using a field validation test by placing five GPS devices on fixed poles (georeferenced using a GNSS GPS device) within the study area, collecting 6,500 positions per static device. The analysis revealed the accuracy error yielded by the devices (mean: 16.4 m, median: 12.6 m). This error was considered in several of the further analyses (see below).

### Satellite imagery

We analysed the spatiotemporal grassland dynamics in the study valley by using satellite images. Given the high spatial and temporal resolution of our tracking data (see the previous section), we sought satellite imagery with comparable resolution. After evaluating different satellite options, we selected the Sentinel-2 mission. The Sentinel-2 satellites, with a nominal altitude of 786 km and a sun-synchronous polar orbit, are designed for the study of land and oceans (e.g., vegetation and soil mapping). Sentinel-2 A and 2B follow sun-synchronous orbits, meaning they pass over the same point on Earth at the same local time, ensuring consistent lighting conditions for image comparisons, benefiting from the use of the same sensor, the Multispectral Imager (MSI). Each satellite has a nominal revisit time of 10 days; however, due to the presence of two satellites in complementary orbits and the “orbit overlap” at mid-latitudes, such as in the study area, the effective temporal resolution can increase to one image every 2–3 days (S2 Mission, n.d).

The first step involved filtering datasets covering the study periods (mid-May to mid-November) for the three years of study (2021–2023). Harmonised Sentinel-2 Surface Reflectance Datasets were processed using Google Earth Engine (GEE hereafter)^36^. One of the main challenges with satellite imagery in the study area is the frequency of cloud cover, which can result in incomplete images and consequently, irregular time series with temporal gaps during cloudy periods, as well as spatial gaps within images. To address this, the Cloud Score+ S2 HARMONIZED dataset was used to mask clouds, followed by a gap-filling procedure^37^. Missing data were filled using pixel-wise interpolation in GEE, where images within a 20-day window were identified using temporal joins and filtered based on time differences. This approach ensured a consistent temporal frequency (2–3 days per image) and reduced cloud-related gaps. Additionally, median composites (at the pixel level with a 10 m spatial resolution) were generated for 10-day non-overlapping periods, typically combining 3–4 images. These composites further reduced noise caused by atmospheric artefacts. To ensure temporal comparability with data on cow movement and minimise early-season cloud gaps, we retained 15 consecutive 10‑day composites per year (45 in total), covering mid‑June to early November (Supplementary Table S1).

Once we obtained the 10-day median Sentinel-2 composites, we calculated vegetation indices such as the Normalised Difference Vegetation Index (NDVI), the Soil-Adjusted Vegetation Index (SAVI), and a moisture index, NDMI (Normalised Difference Moisture Index)^38–40^. SAVI is designed to account for and correct the influence of soil brightness, particularly in areas with sparse vegetation cover. By measuring vegetation greenness and density while minimising the effect of soil reflectance, SAVI provides more accurate vegetation assessments in low-cover regions. This correction makes SAVI especially useful in areas where soil brightness could compromise the accuracy of traditional indices like NDVI. Notably, SAVI has been shown to more effectively identify degraded areas, such as those impacted by grazing, compared to NDVI^41^. Moreover, given the high correlation among the three indices, we selected the Soil-Adjusted Vegetation Index (SAVI) with a soil adjustment factor (L) of 0.5, which is appropriate for areas with intermediate vegetation cover.

The SAVI composites were then downloaded and clipped to the study area using a mask created through photo-interpretation of the 100% Minimum Convex Polygon (MCP) of most tracked cow positions, excluding side valleys and focusing solely on the Catllar Valley. The lower portion of the ridgeline was also excluded. This step was performed using QGIS 3.33^42^. Subsequently, a point feature file was created with points representing the centroid of each SAVI pixel, allowing for the later extraction of data such as topographic variables and variables related to cattle activity that could be associated with SAVI values for each pixel. Once each pixel centroid in the study area was represented by a point, a numerical sequence was generated to arrange the points in a grid from left to right and top to bottom. The final dataset comprised ca. 62,500 pixels with their unique numeric identifier (ID). Finally, the pixel ID and coordinates were extracted to work with spatial points and overlay the points with different rasters.

### Drivers of grassland dynamics

To structure subsequent analyses, we grouped environmental drivers of spatiotemporal grassland dynamics in each pixel into four categories: vegetation type, topography, climate, and cow activity. Within the temporal windows of our study, the first two were time-invariant, whereas the latter were dynamic over time. Topography and climate were considered abiotic drivers, whereas vegetation type and cow activity were considered biological drivers. Extraction of environmental variables and spatial analyses were performed with QGIS and the R packages *raster*^43^ and *sf*^44^.

#### Vegetation types

Because the study focuses primarily on grassland dynamics, we concentrate mainly on grasslands as the primary foraging resource for cows and, secondarily, on shrublands, where grasslands may also be patchily present. These two covers represent most of the surface of the habitat in the valley (see Results). We generated a highly detailed vegetation map of the study area^45^ through the following procedure. First, vegetation patches were photo-interpreted over a 1:2.500 infrared orthophoto taken in 2019^46^. The minimum area of the polygons was set to 2000 m^2^ to ensure that mapped units corresponded to ecologically coherent vegetation patches at this scale. The vegetation types were assigned to a habitat type in the Habitats Manual of Catalonia^47,48^, which is an adaptation of the European CORINE Biotopes Manual^49^ for the Catalan territory and includes about 630 terrestrial habitat types. In the summer of 2020, coinciding with the phenological maximum of the vegetation, fieldwork was carried out in the whole valley to label each polygon with a habitat type and to merge or differentiate new polygons based on field observations. The last phase was the definitive delineation of the polygons, considering the observations made in the field and with the help of complementary cartography (e.g. topography, LIDAR, geology).

To reduce the number of categories and group vegetation types with similar growth cycles and greenness (relevant for SAVI), and to merge vegetation types with minimal representation in terms of pixel count, we performed a reclassification of the vegetation types of interest (grasslands or shrublands) (Supplementary Fig. S2), which resulted in eight categories (six grassland and two shrubland types). We then focused on the centroids of the pixels (see above in section “Satellite imagery”) that correspond to these vegetation types, resulting in ca. 50,500 pixels (10 m spatial resolution). Thus, each centroid of the pixel is ultimately associated with a reclassified vegetation cover type.

#### Topography

To quantify the topographic context shaping vegetation and cow space use, we considered the following topographic variables: elevation, slope, curvature, hydrology (see below), and cardinal orientation (i.e. Digital Aspect Model (DAM), Supplementary Fig. S1). We first downloaded the Digital Elevation Model (DEM) with a 15 m spatial resolution from the Institut Cartogràfic i Geològic de Catalunya (ICGC), the official mapping agency of Catalonia^50^. From the DEM, we derived the Digital Slope Model (DSM) and DAM using QGIS, and the Digital Curvature Model (DCM) using MiraMon^51^, all at 15 m resolution. Hydrology was calculated by creating a raster layer based on the ICGC Hydrography vector data^52^, which maps the small headwater streams and natural springs of the study valley, to measure distance to water sources, and test its effect on cow activity and grassland dynamics (Supplementary Fig. S1). These rasters were then overlaid with the centroid of each SAVI pixel representing grassland or shrubland to extract variable values for analysis.

To reconcile the mismatch between 10 m SAVI pixels and 15 m topographic data and to accommodate GPS positional error, we applied a spatial buffering method. For each SAVI pixel centroid, we constructed a 15 m square buffer that encompassed the central pixel plus its eight immediate neighbours (a 3 × 3 window, ca. 30 × 30 m). Cow GPS positions falling within this buffer could be attributed to any of those nine pixels. This approach reduced spatial misalignment induced by GPS error (see above) and alleviated geometric displacement in mountainous satellite imagery. Because we later subsampled pixels to reduce spatial autocorrelation (see below), we consider that small-scale differences between 10 m and 15 m resolution did not bias our analyses.

#### Climate

To characterise climatic conditions potentially influencing vegetation and cow activity, the study valley is equipped with a weather station that records various meteorological variables on an hourly basis. The station is located at 1450 m a.s.l. in the centre of the valley, within its low altitude range, on a relatively gentle slope and oriented to the south-west (Supplementary Fig. S1). The collected data includes air temperature and relative humidity (mean, maximum, and minimum), land surface and dew point temperature (mean), and solar radiation (mean, maximum, and total). Soil data includes mean water content and conductivity. Wind data includes mean and maximum wind speed (m/s), and precipitation as daily accumulated rainfall.

Given the use of 10-day SAVI composites, we first calculated daily means for all variables but precipitation, which was summed over each 10-day window. We then aggregated these values into 10-day means for the intervals matching SAVI composites. This analysis was conducted at the valley level rather than at the pixel level. To reduce the number of climate variables and assess their collinearity, we used a Pearson correlation matrix and selected one of the two variables that showed correlation coefficients higher than 0.75 (Supplementary Fig. S3). This process resulted in the selection of six climatic variables (abbreviations used hereafter in parentheses): mean relative humidity (RH), mean temperature (Temp), maximum solar radiation (Rad), mean wind speed (Wind), cumulative precipitation (Rain), and mean H_2_O soil content (Soil_H2O).

#### Cow activity and spatial distribution

Because herbivores affect vegetation primarily through grazing and trampling, we focused on distinguishing these two activities. Accordingly, we classified cow activity using accelerometer data retrieved from the tracking collar, specifically the quadratic sum of the standard deviations (QSSD) from the three accelerometer axes (x, y, z)^53^. The quadratic sum approach is advantageous as it assigns greater weight to larger values, which are indicative of higher activity, while minimising the impact of smaller values, thus enhancing the ability to distinguish between activity and inactivity.

To define activity thresholds, we performed exploratory analyses on data from the static GPS devices (Supplementary Table S2). We defined the thresholds as follows: “Resting”: QSSD < 388 (which captured nearly 100% of the data from static GPS devices), “No Class”: 388 ≤ QSSD ≤ 940 (a range associated with potential classification uncertainty) and “Grazing”: QSSD > 940 (as no values above this threshold were recorded by static devices). Moreover, to refine the classification further, we applied a temporal correction to “No Class” positions. Short bouts of grazing or resting are common in cattle, and ‘No Class’ values typically arise at the transitions due to accelerometer ambiguity. Therefore, if a “No Class” position was flanked by positions of the same activity (either grazing or resting) within a time gap of less than 15 minutes, it was reclassified to match the adjacent activity to reduce edge‑misclassification. For “Resting”, we imposed an additional condition: the distance to either the previous or subsequent position had to be less than 30 m for reclassification as “Resting”. This refinement process resulted in a reduced number of “No Class” positions and improved activity classification into two categories: grazing and resting. To qualitatively validate classification outputs, we conducted field observations of cow behaviour during the grazing season (N = 157 hours of observations), which confirmed that accelerometer‑derived “grazing” and “resting” categories matched the behaviours observed in the field.

To relate cow activity to vegetation dynamics, we associated SAVI pixel values (10-day medians) with the number of grazing and resting positions falling within each pixel during each 10-day interval. This method addresses two key sources of noise: (1) reducing the possible error associated with GPS data and (2) mitigating the geometric displacement present in satellite imagery (99.8% of pixels in mountainous areas exhibit DEM-induced displacements within ± 10 m)^54^.

For each 10-day interval between SAVI composites, we calculated the number of grazing and resting positions for every SAVI pixel. Additionally, the total number of positions for each pixel was divided by the number of GPS devices deployed each year (80 for 2021 and 2022, and 85 for 2023). Only SAVI pixels corresponding to grassland or shrubland vegetation types (~ 50,500 pixels) were included in the analysis.

To avoid attributing grazing to resting-dominated patches, we applied a filtering procedure. For each year, pixels identified as resting areas (resting ≥ 1, i.e. at least one resting fix.

in any 10-day interval) were assigned a grazing value of 0, ensuring these areas were treated as resting rather than grazing patches. By doing this, we removed locations where cows were largely inactive (e.g., standing, resting, or ruminating) from being interpreted as grazing activity.

To analyse the spatial distribution of cows and test for their clustering, we applied Ripley’s K function at the herd level using all fixes pooled across tracked cows and the Pair Correlation Function *g*(*r*). The distance parameter *r* represents the spatial scale at which point interactions are evaluated. Ripley’s K function examines the degree of spatial dependence by comparing the observed distribution of points (GPS fixes) to a null model of complete spatial randomness (CSR), with K(r) values above expectation indicating clustering. The Pair Correlation Function *g(r)* provides a complementary, scale‑specific measure by identifying specific spatial scales where clustering occurs, providing a localised measure of point interactions. Statistical significance of deviations from CSR was evaluated using Monte Carlo simulations, allowing us to determine whether cows exhibit habitat selection and social aggregation.

### Statistical analysis for SAVI dynamics

To ensure reliable inference, we first checked for autocorrelations in SAVI data and time legacies in dynamic explanatory variables. We considered these two potential sources of bias to build reliable regression models that explain the effects of explanatory variables on spatiotemporal SAVI dynamics.

#### Temporal and spatial autocorrelation

Because vegetation indices time series and maps commonly exhibit temporal and spatial dependence, respectively, we tested the occurrence of temporal and spatial autocorrelation using the Partial Autocorrelation Function (PACF) to assess the temporal and spatial windows at which autocorrelation was too high. We considered PACF = 0.5 to be a threshold for explanatory lags. Regarding temporal autocorrelation, we analysed how many past time lags (of 10 days) explained the observed SAVI at a given time. The results indicate that the first time lag, specifically the 10-day median SAVI just before the target SAVI median, correctly explains the temporal autocorrelation (Supplementary Fig. S4).

We analysed spatial autocorrelation in SAVI values by using concentric windows around each central pixel of interest. The first window (Window 1) represents the average SAVI value of the 8 pixels surrounding the central pixel. The second window (Window 2) calculates the average SAVI of the 16 pixels surrounding Window 1, and subsequent windows progressively expand to encompass additional surrounding pixels. PACF analysis using 5,000 random pixels from two different SAVI images showed that spatial autocorrelation was no longer statistically significant at Window 5 (Supplementary Fig. S5A). However, to adopt a more conservative approach, we chose Window 6 as the threshold window above which autocorrelation faded. Based on this criterion, we selected a random subset of pixels by overlapping a 60 m resolution spatial grid on the SAVI images (which have a 10 m spatial resolution), which eliminated spatial autocorrelation (Supplementary Fig. S5B).

#### Time legacies of dynamic variables on SAVI

We then assessed the potential temporal inertia of the dynamic explanatory variables (i.e. climate and cow activity) on SAVI dynamics. We tested the effect of lag-time windows, starting with an initial 10-day window. We used these variable-specific temporal windows to evaluate our study aims in all subsequent regression models (see the following section). In each subsequent model, we incrementally included the preceding 10-day window, extending the total lag period by steps of 10 days, up to a maximum of 60 days (we chose this limit as a conservative maximum because temporal autocorrelation had effectively disappeared for all variables by that lag). For climatic variables, we computed 10-day means of the daily mean values, except for precipitation, which was the accumulated value over each window. For grazing and resting, the total number of positions within each SAVI pixel was summed and normalised by dividing by the number of active GPS devices deployed during that year, as previously described. We did not test effects within the 10-day window interval, because each SAVI composite already represents the median of 2–4 images taken within that same interval, making sub‑interval testing inappropriate and not comparable to the temporally aggregated covariates. Therefore, although a single SAVI value is used as the response, it originates from several values corresponding to different dates within those 10 days. Conversely, for grazing, resting, or climatic variables, the value obtained represents the accumulated total or average for the 10-day interval. Results from modelling showed that while some variables have an immediate effect of the value in the same 10‑day interval on SAVI (relative humidity, H_2_O soil content, solar radiation and cattle both grazing and resting), others have longer temporal legacies (cumulative rainfall, wind speed and temperature) (Supplementary Table S3). We took these temporal windows specific to each variable for the subsequent regression modelling.

#### Multiple regression modelling

Finally, we addressed the three overarching questions outlined in the Introduction: climate vs. grazing strength, topographic modulation, and grazing nonlinearity. Thus, we used Generalised Linear Mixed Models (GLMM) to test the effects of explanatory variables on spatiotemporal SAVI dynamics using the R package *lme4*. We first standardised all explanatory and response variables before the models were generated. This standardisation ensures that all variables are on the same scale, facilitating model fitting and interpretation. Since our subset of data was corrected for spatial autocorrelation, we included the temporal autocorrelation (specifically the 10-day median SAVI just before the target SAVI median, see above) as a random effect in the models. Before model selection, we reduced dimensionality by removing collinear predictors and retaining the screened set used in candidate models, thus lowering the risk of overfitting. For selecting the best model based on a parsimony principle, we used the Akaike Information Criterion (AIC), and we calculated the weight of each model and the ΔAIC, which is the difference in AIC value between a model and the selected model, i.e. the one with the lowest AIC value. We considered that the two models having ΔAIC ≤ 2 were statistically equivalent. Standard cross‑validation was not applied because spatial and temporal dependence would violate fold independence, and the autocorrelation structure was instead modelled explicitly through spatial subsampling and a temporal random effect. Finally, GLMMs were chosen because we aimed to estimate interpretable effect sizes and hypothesis‑driven interactions within a hierarchical error structure, rather than to optimise predictive performance.

Due to the high number of potential drivers and the number of processes involved, we proceeded with our analyses in four steps (Fig. 1). In the first step, we started with the full model (including all the 24 explanatory variables selected after testing for collinearity, see Results) and analysed all 16,384 possible additive combinations. Curvature and relative air humidity were not retained in the provisionally selected best model and were discarded in the subsequent steps (Supplementary Table S4).

In the second step, we compared the time-varying model of SAVI with a model for each grouped variable (vegetation types, topography, climate, and cow activity) to assess the relative contribution of each group to the variance in SAVI. This approach is aligned with our broad aims of assessing how grazing intensity and climatic drivers influence spatiotemporal SAVI dynamics, and of comparing the relative importance of these drivers. We used the analysis of deviance (ANODEV) procedure to evaluate how the inclusion of a specific grouped variable improved the model fit by comparing the deviance (a measure of model fit) between models with and without the variable^55^. ANODEV of each model was calculated as follows:

$$\:ANODEV\:=\:\frac{Dev\left(X\right)-Dev\left(constant\right)}{1}/\frac{Dev\left(time\right)-Dev\left(X\right)}{K-1}$$

where Dev(constant), Dev(*X*), and Dev(time) represent the deviance values of the null model (time-invariant model), variable-dependent model, and time-dependent model, respectively, and *K* is the number of parameters in the model. The time-dependent model has an ANODEV value of 1, while ANODEV values show the amount of temporal variation explained by variable *X*. Consequently, the null model has an ANODEV of 0.

In the third step, we tested our expectation regarding about non-linear associations between grazing and SAVI. We built a model with a quadratic relationship and a model with a log association, which would also test a nonlinear change of vegetation by grazing, but up to a threshold, beyond which additional grazing had minimal impact. Among the three models tested, the best was that with a log negative influence of grazing on SAVI dynamics, whereas the quadratic and the linear models, although also showing negative relationships, performed worse (ΔAIC = 161 and 231, respectively). The log relationship corresponded to a steep initial decline in vegetation response to grazing, followed by a diminishing negative effect as grazing intensity increased. Thus, we selected the log shape of grazing in the subsequent analysis.

In the final step, we focused on our third broad question, i.e. whether cow activity (with a log pattern, see above) interacted with static variables (i.e. topography and vegetation types) to influence spatiotemporal SAVI dynamics. We tested whether these two models (one for grazing and one for resting) performed better than a full model with the selected variables from the previous analysis (i.e. the additive model‑selection step) and without interactions. We preferred to use climatic variables from a single station located in the valley because these data have higher temporal resolution than those obtained from commonly available gridded climate model datasets^56,57^. Because climatic drivers were only available at the valley scale, we did not test for interactions between climate and other predictors, especially cow activity. We acknowledge that this scale mismatch (valley-level climate data versus pixel-level SAVI and the rest of the drivers) may limit the resolution of estimated climate effects, while topography (especially altitude and orientation) would partly reflect the microclimatic variability that we could not capture with the available climate data. This should not affect rainfall, which was likely the same for the whole valley, since in the Pyrenees, most summer rainfall is produced by orographic stratiform and moderate convection, not by strong isolated cells.

## Results

### Cow activity

We obtained a total of ~ 465,500 fixes (classified as grazing or resting) from tracking over the three seasons, after applying a random selection of pixels to correct for spatial autocorrelation. A notable percentage of pixels did not record any cow fix for at least one year (36.9%) or for at least two years (19.4%), while only 2.1% did not record any cow fix over all three years.

**Fig. 3:**
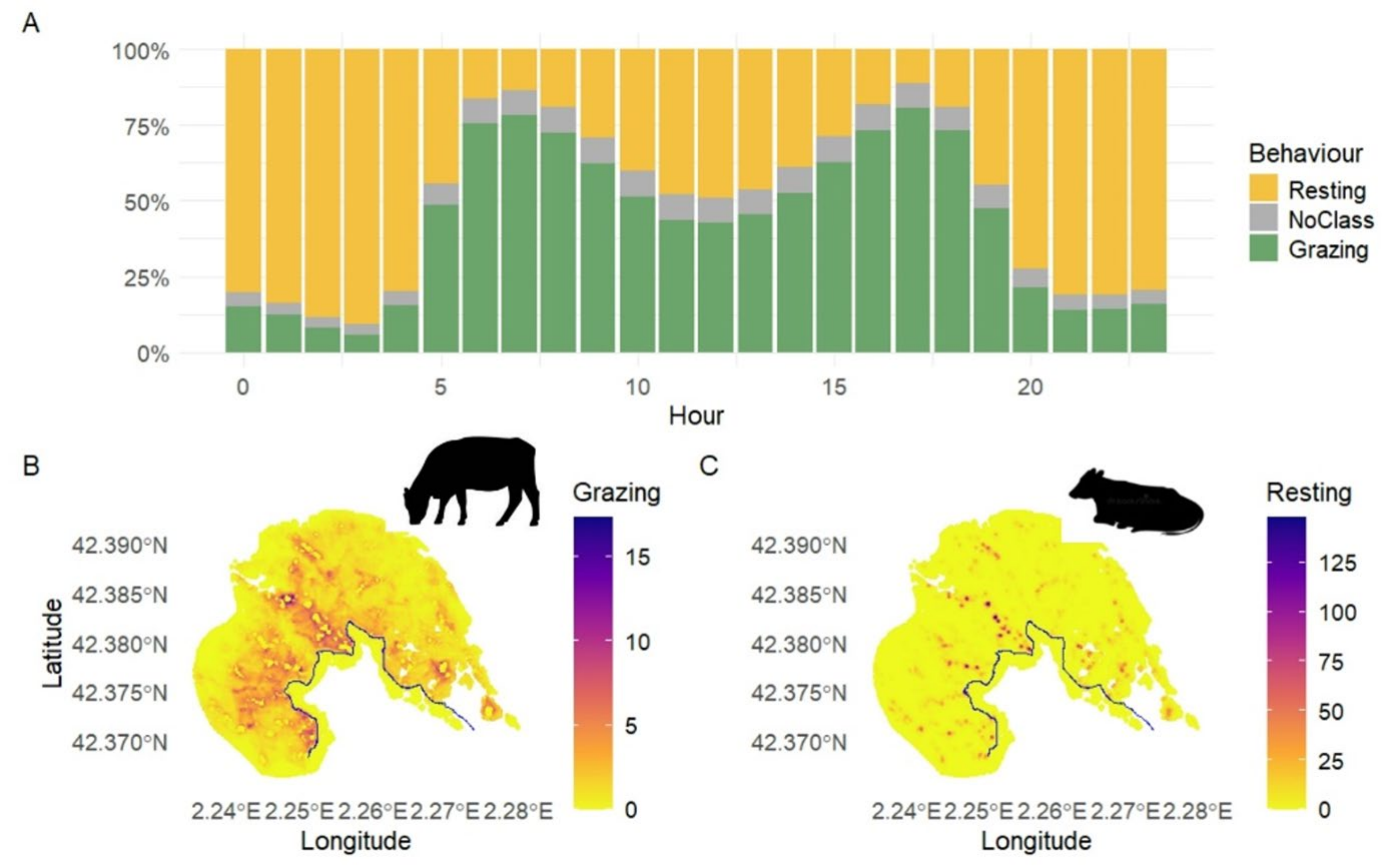
**(A)** Circadian activity patterns of cows, represented as the percentage of grazing, resting, or ‘No classified activity’. Data are based on the total GPS positions of all individuals combined across the three study years. Hours are + 2 UTC. (**B** and **C**) Spatial distribution of cow activity in the study valley for grazing and resting respectively, across seasons, aggregated per pixel. The values represent the number of positions accumulated, normalised by the number of GPS devices deployed, for each 10 m pixel. The figures display the centroids of the studied pixels. Images also show the position of the electric fence (blue line), limiting the movement of cows to the upper half of the valley. The seasons span from early July to late October. Geographic coordinate system, Datum WGS-84 (EPSG:4326).

When we analysed cow activity, we observed a higher proportion of “Resting” positions during observed resting hours (either at midday or at night), and “Grazing” positions were more prevalent when cows were more active (see Fig. 3A). Grazing and resting occurred across all vegetation types, with grazing more frequent in productive grasslands. Nevertheless, resting was restricted to particular patches, and results suggest that grazing occurred mainly around those resting patches (Fig. 3B-C). This matches with both our QSSD-based dichotomy for cow activity classification and our field observations. Cows showed a clumped spatial pattern, far from a random distribution and similar in all three years of study (Supplementary Fig. S6). The observed *K(r)* fell outside the Monte Carlo simulated envelope, which showed a statistically significant effect of clustering (significance level of pointwise Monte Carlo test: 2/100 = 0.02).

### SAVI seasonal dynamics

**Fig. 4:**
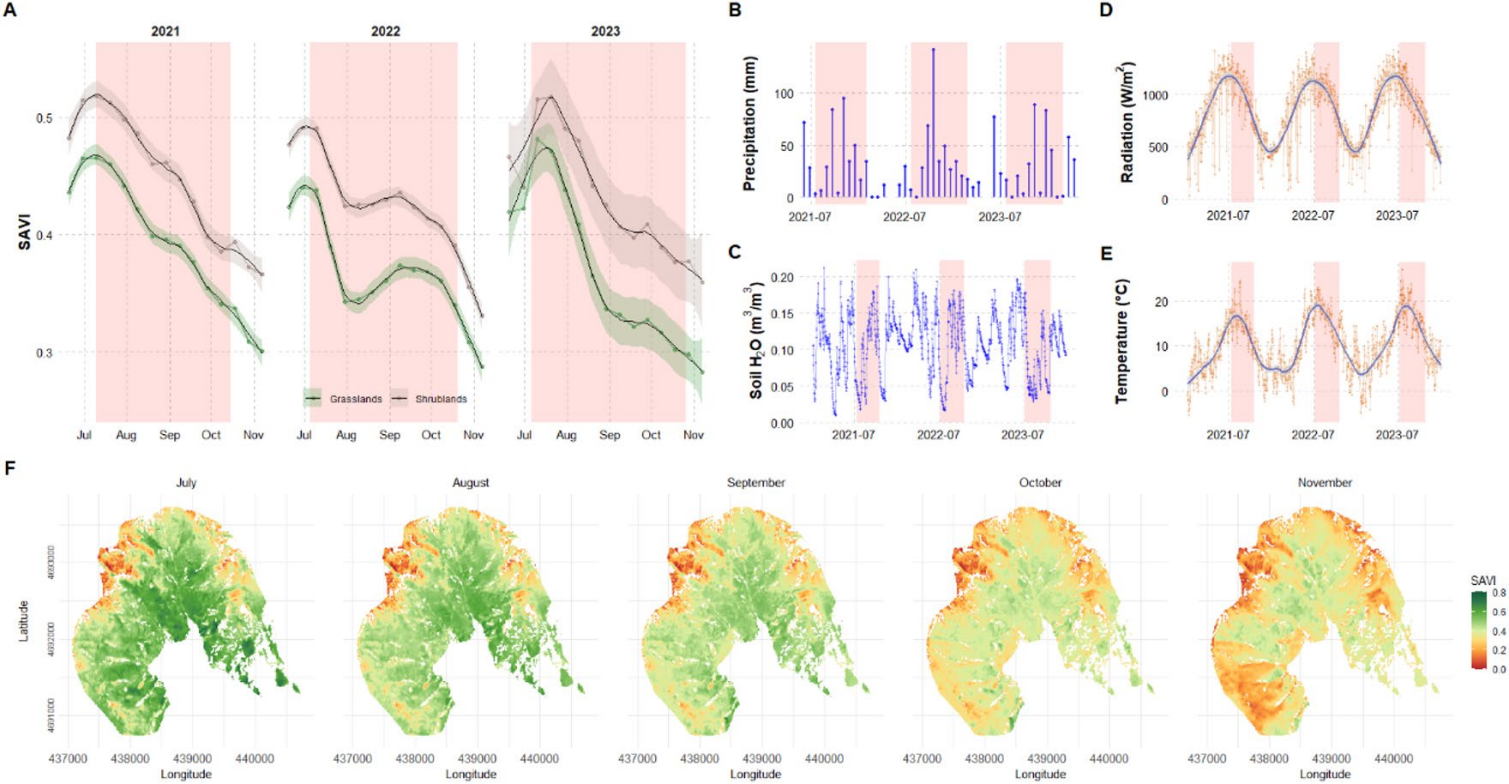
**(A)** Seasonal cyclic dynamics of mean SAVI index for the two main vegetation types (grasslands and shrublands) over the three study year seasons. Dynamics among these seasons cannot be shown since the valley is mostly covered by snow and SAVI could not be estimated. We show non-seasonal (rainfall **(B)** and water soil content **(C)**) and seasonal dynamics of climate variables (solar radiation **(D)** and temperature **(E)**) over the same study period. Pink bars indicate the grazing seasons of the cows. Lines and shadows represent the mean and standard error, respectively, of a LOESS regression model fitted to the data. Soil volumetric water content is expressed in cubic metres per cubic metre (m³ m⁻³), solar radiation in watts per square metre (W m⁻²), rainfall in millimetres (mm), and air temperature in degrees Celsius (°C). **(F)** Spatiotemporal dynamics of SAVI for each month of the 2021 grazing season, as an illustrative year (spatiotemporal patterns were similar across years). Projected coordinate system: ETRS89/UTM zone 31 N (EPSG:25831).

The SAVI dynamics showed a seasonal pattern, with cattle entering the study area precisely when the grasslands were greenest (i.e. highest values of SAVI, Fig. 4A). Overall, SAVI declined through the season, with pronounced fluctuations in 2022 (Fig. 4A), likely driven by a positive rainfall anomaly (Fig. 4B). Likewise, the strong seasonal cycle of SAVI (Fig. 4A, F), peaking in early summer and declining toward winter, is in line with the well‑established influence of solar radiation and temperature during the warm growing season on vegetation greenness (Fig. 4C-E), and this seasonal decline was further accentuated in areas of higher grazing use (see also Fig. 3).

### Relative explanatory importance of each environmental variable in the spatiotemporal dynamics of SAVI

**Fig. 5:**
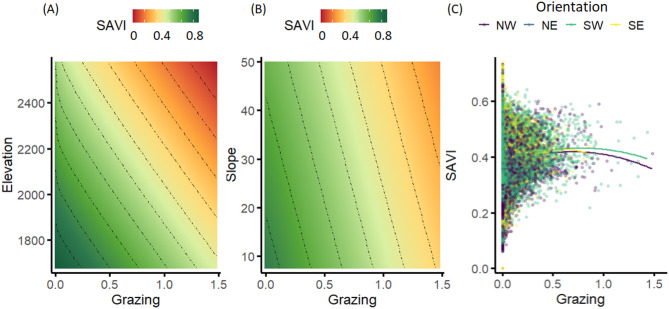
Interaction between grazing intensity (measured as log‑scaled, standardised fix counts per pixel) and topographic variables that were selected in the modelling of SAVI dynamics. **(A)** and **(B)** panels show a contour landscape of how SAVI varied with the interaction of grazing with elevation and slope, respectively, using the estimates of the finally selected model. The steepness and orientation of the contour lines reflect how the marginal effect of grazing varies along topographic gradients to influence SAVI spatiotemporal dynamics: the steeper gradient in panel **(A)** compared to panel **(B)** indicates that elevation modulates the grazing effect more strongly than slope. **(C)** SAVI responses to grazing for the four orientations occurring in the study valley (NW, NE, SW, SE); here, lines represent a LOESS regression fit (note that we only had 135 pixels for NE orientation, thus its fitted line is omitted for clarity, compared with the range of data for the other three orientations; range: 13185–30105 pixels). Note that the absolute magnitude of the grazing and orientation effects is small relative to overall SAVI variability.

**Fig. 6:**
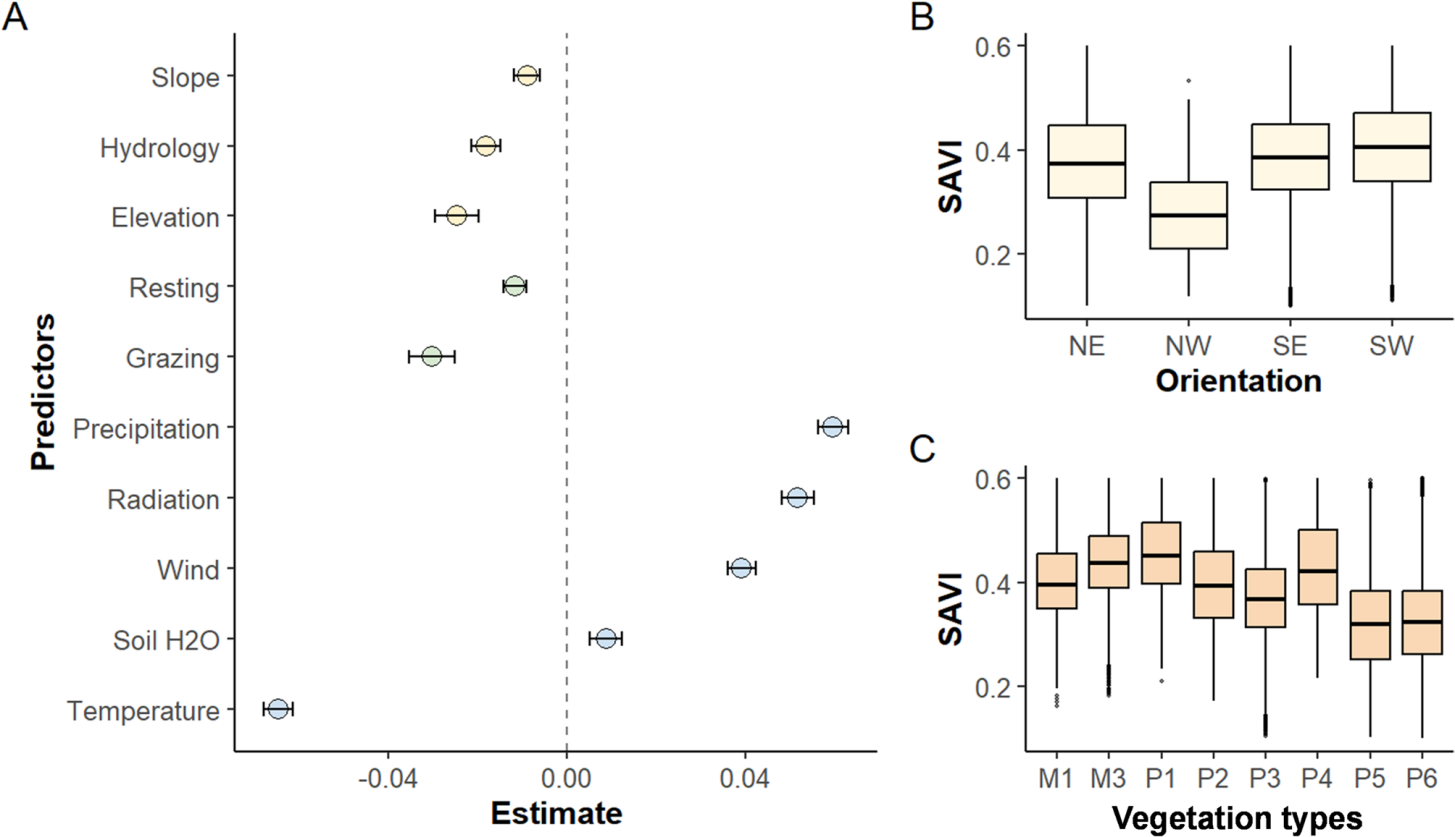
**(A)** Estimated coefficients from the selected linear mixed model predicting SAVI based on standardised predictor variables (Supplementary Table S4). Grazing includes the combined effect of both the linear and logarithmic terms. Points represent estimated coefficients, while horizontal bars indicate 95% confidence intervals. For categorical variables, boxplots are shown instead, representing the raw distribution of empirical SAVI data used to fit the model with orientation **(B)** and vegetation types **(C)**. These types are: P1 *Festuca nigrescens - Agrostis capillaris* (mesophilous grasslands); P2 *Nardus stricta* (tussock mesophilous grasslands); P3 *F. eskia* (upper subalpine–low alpine grasslands); P4 *F. paniculata* (grasslands on deep soils, warm slopes); P5 *F. airoides* (alpine grasslands); P6 *F. gautieri–F. yvesii* (dry, rocky slope grasslands). Details about units of each variable are further explained in Methods and Appendix S1 (Fig. S2).

As we noted in the Methods, we first found that all variables but curvature and relative humidity were retained in the provisional best model (Supplementary Table S4). The explanatory variables grouped in the four categories (climate, cow activity, vegetation type and topography) accounted for 46% of the temporal variability of SAVI (Table 1). Among these variables, climate, cow activity, vegetation types, and topography explained 83%, 12%, 3% and 2% of that variability, respectively. Thus, climatic drivers were ca. an order of magnitude more influential than cattle activity in explaining temporal variation in SAVI (38% versus 5% of explained temporal variability when assessed independently; Table 1). Climate showed the best statistical support, reflecting its close correspondence with the pronounced seasonal pattern observed in SAVI (Fig. 4). Cow activity had a negative effect on vegetation (with grazing having a stronger impact than resting), supporting our expectation that intensive grazing reduces vegetation greenness (Figs. 5 and 6).

**Table 1:** Model selection for the explanatory variables of SAVI dynamics grouped by the four categories. ‘Time‑varying’ and ‘Constant model’ refer to models including temporal variation and intercept‑only models, respectively. Np = number of parameters of the model; AIC = Akaike Information Criterion, ΔAIC = difference of AIC value relative to the best model; ANODEV = amount of temporal variability in SAVI explained by each model (see Methods). Models are ranked by their AIC descending value, and the selected model is in bold. The category ‘Cow activity’ encompassed grazing (quadratic shape) and resting.

Model	Deviance	np	AIC	ΔAIC	ANODEV
**Time varying**	**27,057**	**45**	**27,496**	**0**	**1**
Full model	38,087	22	38,343	10,847	0,46
Climate	39,628	6	39,703	12,207	0,38
Cow activity	46,289	4	46,334	18,838	0,05
Vegetation type	47,126	8	47,211	19,714	0,01
Topography	47,208	7	47,287	19,791	0,01
Constant model	47,392	1	47,401	19,905	0

### Interactions between drivers in the spatiotemporal dynamics of SAVI

The broad question we addressed here was to what extent topography modulated the effects of grazing and climatic drivers on spatiotemporal SAVI dynamics. Models including an interplay between nonlinear grazing and three topographic variables (elevation, slope, and orientation) performed better than the full model without interactions (Supplementary Table S5). SAVI decreased non-linearly with grazing, and lower SAVI values occurred particularly at higher elevations, steeper slopes, and the patches oriented to northern hillsides (Fig. 5). Although the effects were statistically supported, the magnitude of the grazing‑induced decline in SAVI was small relative to the total variability in the data, and differences among orientations were modest (Fig. 5C). On the contrary, including interactions of resting with topographic and vegetation type variables and grazing with hydrology and vegetation types did not improve the full model.

The selected model (Supplementary Table S4, model 1) also showed that increasing water soil content, accumulated rainfall, solar radiation (which should enhance photosynthesis), and wind, generated an increase of SAVI, whereas increasing temperature decreased SAVI values (Fig. 6). As expected, continuous topographic variables had a negative effect on SAVI, especially elevation and hydrology (i.e. distance to water sources). We found that north-west oriented slopes exhibited the lowest mean SAVI (Fig. 6). Regarding vegetation types, SAVI attained its highest values for the P1 type, dominated by grasslands of the species *Festuca nigrescens* and *Agrostis capillaris*, with patchy-distributed *Genista saggitalis* and lower values in P5 and P6 types corresponding to alpine and subalpine grasslands on slope and stony soils (Fig. 6).

## Discussion

We examined how herbivore activity (both grazing and resting), climate, and topography jointly shaped grassland dynamics in a subalpine Pyrenean ecosystem during the 2021–2023 grazing seasons (summer–autumn). We focused on extensively managed cows, the dominant large herbivore in many mountain grasslands. As in other systems, cows used space heterogeneously at fine spatial scales. Within the patchy structured vegetation mosaic largely determined by topography, grazing produced additional local declines in SAVI along environmental gradients. Our results show that both grazing (consumptive) and resting (non-consumptive) activities reduced SAVI, with grazing producing the strongest overall decline, whereas resting caused more localised (i.e. recursive resting patches), recurrent impacts consistent with trampling and nutrient/urine deposition. Higher grazing intensity was associated with lower SAVI, but the relationship was non-linear, with the strongest marginal decline occurring at low grazing intensity, contrasting with studies reporting facilitative effects at similar levels^58,59^. Rainfall increased SAVI, while temperature shaped its seasonal dynamics. Together, these results indicate that climate primarily structures the seasonal dynamics of SAVI, whereas spatial patterns reflect the combined influence of environmental gradients (particularly elevation) and herbivore activity. By contrast, topographic effects were weaker and partly inconsistent with patterns recorded in other mountain systems (e.g. orientation), suggesting that local microclimate and site-specific factors (e.g. elevation, soils, vegetation type) may be more influential^60,61^.

### Seasonality of spatiotemporal vegetation dynamics

We observed a strong seasonal pattern of SAVI, consistent with grasslands globally^62^ and in mountain ecosystems at similar latitudes^63,64^. SAVI peaked in early summer when cows entered the valley and declined towards winter as grasslands became dormant. Climate variables with strong seasonality, particularly solar radiation and temperature, showed high relative explanatory importance for SAVI, while non-seasonal variables (e.g. soil water content, accumulated rainfall, wind) also contributed to explained variation. This pattern reflects the life cycle of subalpine plants, which peak in productivity in late spring and decline as rising summer temperatures and water stress constrain growth. This observation aligns with traditional ecological knowledge (TEK), where local herders often rely on understanding vegetation cycles and climatic patterns to determine optimal grazing times, a pattern also documented in other Pyrenean transhumance systems^65,66^. The positive association between SAVI and rainfall/soil moisture suggests that moisture inputs can buffer vegetation against summer drought and extend active growth^60,67^. Time lags differed among variables: SAVI responded most strongly to recent soil water content and solar radiation, whereas temperature, wind, and accumulated rainfall showed longer lagged effects, consistent with delayed grassland responses to persistent climatic conditions^68–71^.

### Movement of cows and their effects on vegetation dynamics

Large herbivores influence grasslands through defoliation, trampling, and patchy nutrient return, with outcomes contingent on evolutionary grazing history and environmental context^59,72,73^. Grasslands have a long evolutionary history of mammalian grazing and often show compensatory growth after defoliation^58,73,74^. At the same time, foraging behaviour is shaped by interactions between herbivore traits, plant traits, and seasonal changes in forage quantity and quality^75^. As plants transition from vegetative growth to flowering and senescence, biomass, greenness, and nutritional quality decline, increasing rumination time and promoting shifts in patch use^76^.

In our system, reduced SAVI on steep and south-facing slopes is consistent with harsher microclimates (higher irradiance, lower soil moisture), although a commonly observed pattern of higher SAVI on north‑facing slopes was not strongly expressed in our data. This suggests that the influence of microclimatic environments may be partially overridden by grazing intensity, vegetation structure, or local site conditions^60,61,67^. Interestingly, SAVI did not show strong interannual trends, suggesting that grazing and resting impacts are largely regenerated each year, consistent with a heuristic harvesting based on TEK and extensive management practices^66^.

Cows moved collectively and non-randomly, producing clumped spatial distributions and heterogeneous grazing pressure. This patchy spatial use also reflects the heterogeneity of the landscape, where grassland patches suitable for grazing are scattered with steep slopes, rocky patches, or otherwise non-preferred plant species (e.g. shrubs such as *Juniperus* spp. or toxic forbs such as *Aconitum* spp). As a consequence, large parts of the valley remain lightly used or unused by cattle, a pattern typical of low-intensity pastoral systems where herbivores concentrate their activity in the most productive and accessible patches^77,78^. Although vegetation types differed in their overall greenness (i.e. reflecting contrasts in productivity and soil conditions), the reduction in SAVI with increasing grazing intensity was broadly similar across types. This suggests that, despite ecological differences among communities, herbivory acts as a comparatively uniform pressure on canopy greenness in this subalpine ecosystem. Recursion to specific resting patches, which are scarce and associated with gentle slopes and open areas, led to intensive seasonal use^21,79,80^. Interestingly, this recursion constrained grazing to be more intense in the immediate surroundings of the resting sites, creating halos of high use where animals alternated between resting and foraging. Such recursive movements also generated localised but recurrent impacts on vegetation and support previous results on the effects of trampling and dung/urine deposition on soil structure and vegetation^11–13,81^.

Regarding grazing, SAVI declined non-linearly with grazing relative intensity, with a steep initial decrease followed by a flattening of the response. At low to moderate intensities, rapid removal of palatable biomass reduces greenness detectable by vegetation indices. At higher intensities, remaining vegetation consists mainly of short stubble or less palatable species, a pattern reported under heavy grazing pressure, constraining further SAVI declines^58,73^. Oral morphology limitations (cattle cannot graze below a minimum stubble height because grass must be grasped with the tongue) and compensatory growth responses may further buffer additional losses^58,73,74,82^. These mechanisms together can produce stronger marginal declines at low grazing intensity, although part of this early drop may also reflect the contrast between ungrazed and lightly grazed areas, a pattern described in other grazing studies^30,31,58,73^.

### Limitations of our study

Although our study provides new insights into the relative contributions of grazing, vegetation types, and abiotic drivers to grassland dynamics, several limitations should be considered. While SAVI is effective for detecting spatiotemporal patterns^26,83^, it cannot resolve finer ecological processes such as plant species composition or Aboveground Net Primary Production (ANPP)^59,73^. Furthermore, we acknowledge that the temporal aggregation of grazing, resting, and climatic variables into 10‑day intervals may not always align perfectly with the temporal dynamics captured by the SAVI composites. This mismatch could introduce temporal noise in the estimated relationships.

Behavioural classification into grazing and resting, based on QSSD thresholds, was not validated with an independent labelled dataset, preventing formal accuracy estimates (e.g. confusion matrices). Nevertheless, field observations and previous studies^79,80^ suggest qualitative agreement. Moreover, this coarse classification cannot quantify key components of grazing intensity (e.g. bite rate, actual biomass removal, or grazing duration)^53^, so dynamics mediated by grazing capacity, duration, and plant selectivity remains unmeasured.

Wild chamois may exert an unquantified influence on vegetation signals; however, their numbers were much lower than those of cattle, and their summer habitat use was constrained by livestock presence, so any bias is likely small.

Finally, climate data were measured at the valley scale and applied at the pixel level, which may underestimate fine-scale microclimatic variation (except rainfall). Consequently, climate effects should be interpreted as reflecting valley-wide influences rather than microhabitat-level conditions (e.g. irradiance or temperature differences with elevation and orientation).

## Conclusions

Vegetation dynamics in subalpine grasslands are shaped by the combined effects of biotic (livestock activity, and differences among vegetation types in baseline SAVI) and abiotic drivers (climate, topography), with strong seasonal periodicity. Grazing intensity exerted a negative, non-linear influence on SAVI, whereas resting produced localised but recurrent impacts consistent with trampling and nutrient deposition. Climate variables with clear seasonality (temperature and radiation) primarily explained SAVI seasonal cycles, while rainfall and soil water content buffered short-term variation. Among topographic features, elevation and slope modulated some effects but played a comparatively smaller role, likely reflecting underlying microclimatic differences (e.g. elevation, orientation). Overall, our results highlight the need to account for both consumptive and non-consumptive livestock activities, and for the joint effects of climate and topography, when evaluating spatiotemporal grassland dynamics.

## Supplementary Information

Below is the link to the electronic supplementary material.

Supplementary Material 1

## Data Availability

Datasets are available with open access (CSIC public repository) at: [http://hdl.handle.net/10261/418133].
