# Supplementary material for: Large herbivores and abiotic drivers jointly shape spatiotemporal grassland dynamics in a subalpine ecosystem
Source: Sci Rep. 2026 Apr 14;16:22278. doi: 10.1038/s41598-026-45843-0 (PMC13373178; doi:10.1038/s41598-026-45843-0)
Supplement: Supplementary file 1 — Supplementary Material 1 [file 41598_2026_45843_MOESM1_ESM.docx]

***Supplementary Information***


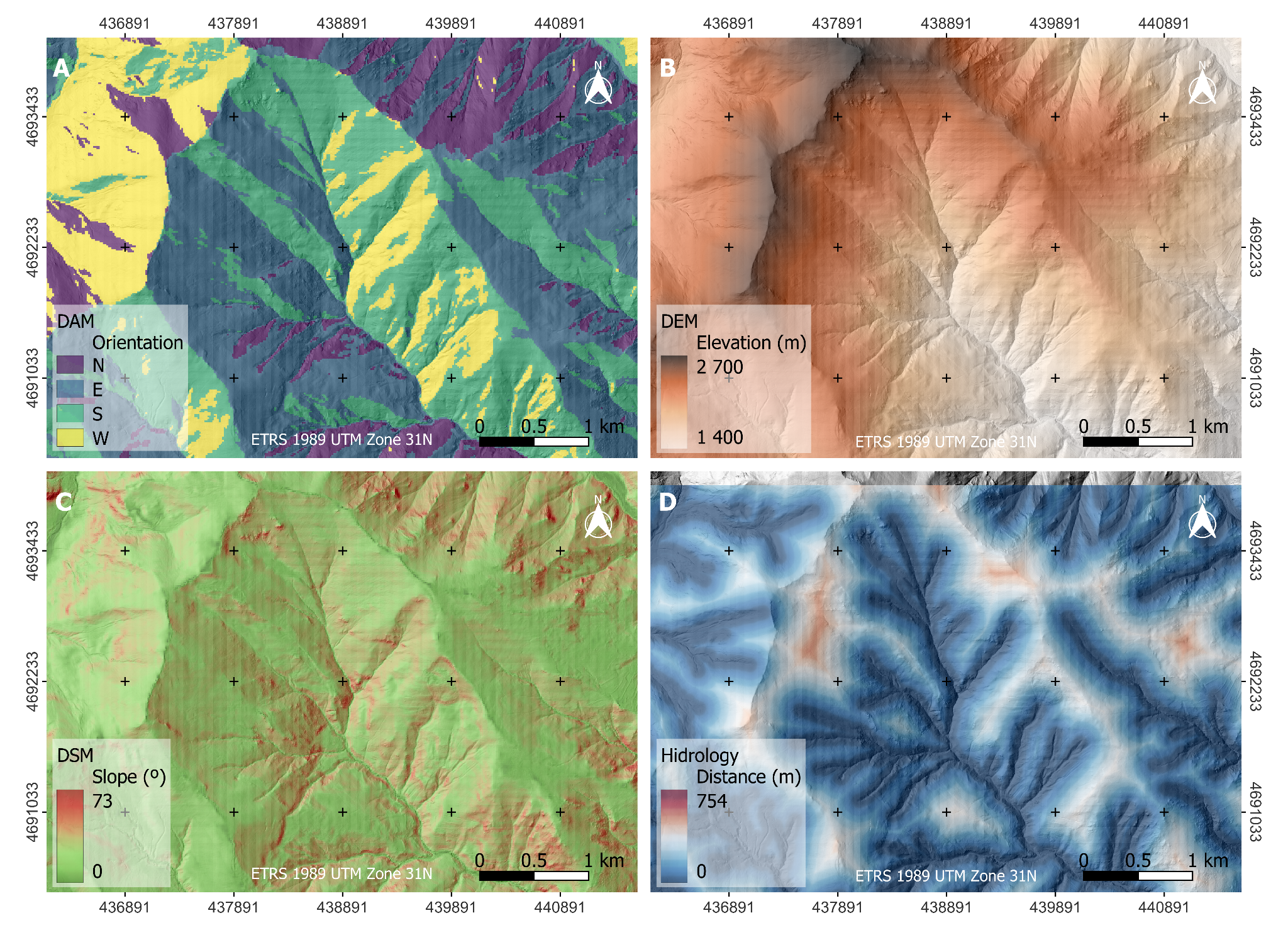


**FIGURE S1**. (A) Digital Aspect Model (i.e. cardinal orientation) with a 15 m spatial resolution, (B) Digital Elevation Model with a 2 m spatial resolution, (C) Digital Slope Model with a 15 m spatial resolution, and (D) Distance to Hydrology with a 1 m spatial resolution. All maps are displayed with transparency and overlaid on a Hillshade Map. The red star shows the location of the meteorological station.


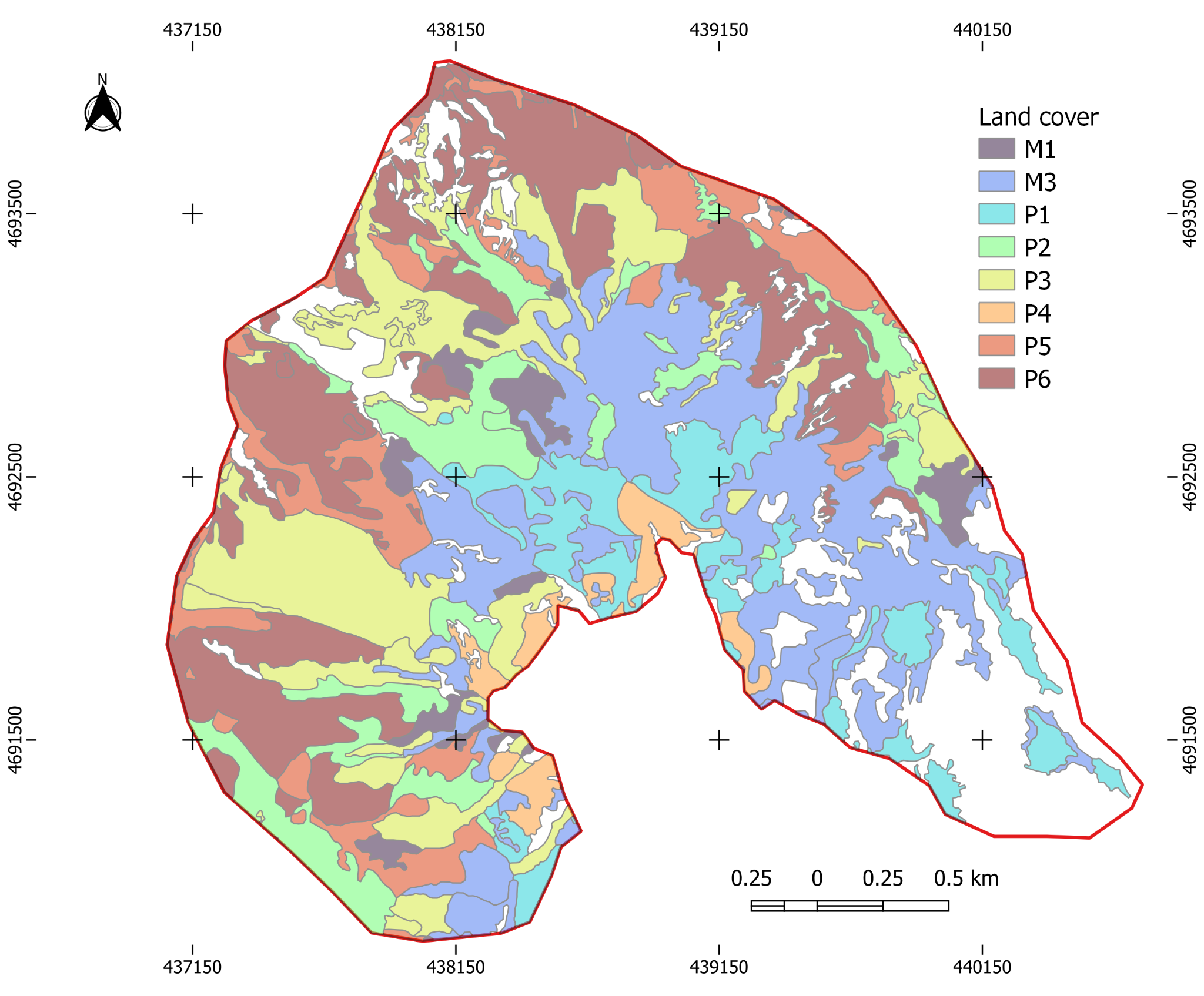


**FIGURE S2**. Reclassified map of vegetation types in the study area, with six categories of grassland and two of scrub. The white areas correspond to habitats that are not of interest: forests, cliffs and screes. The categories in the map (with the original Corine codes in parentheses) are:

M1: Heathlands with *Calluna vulgaris, Vaccinium myrtillus* and *V. uliginosum* (CORINE 31.2261+, 31.412)

M3: Shrublands with *Juniperus nana* and *Cytisus oromediterraneus* (CORINE 31.431, 31.8422)

P1: Mesophile silicicolous montane grasslands with *Festuca nigrescens, Agrostis capillaris* and *Genista sagittalis* (CORINE 35.12)

P2: Mesophile tussock grasslands of *Nardus stricta* (CORINE 36.311)

P3: *Festuca eskia* grasslands or garland-grasslands of the upper subalpine and Iower alpine (CORINE 36.315, 36.332)

P4: *Festuca paniculata* grasslands on deep soils of steep, rocky, warm slopes (CORINE 36.3311)

P5: Alpine grasslands of *Festuca airoides* (CORINE 36.3431+)

P6: *Festuca gautieri* and *F. yvesii* grasslands on dry and stony slopes (CORINE 36.3432+, 36.434)


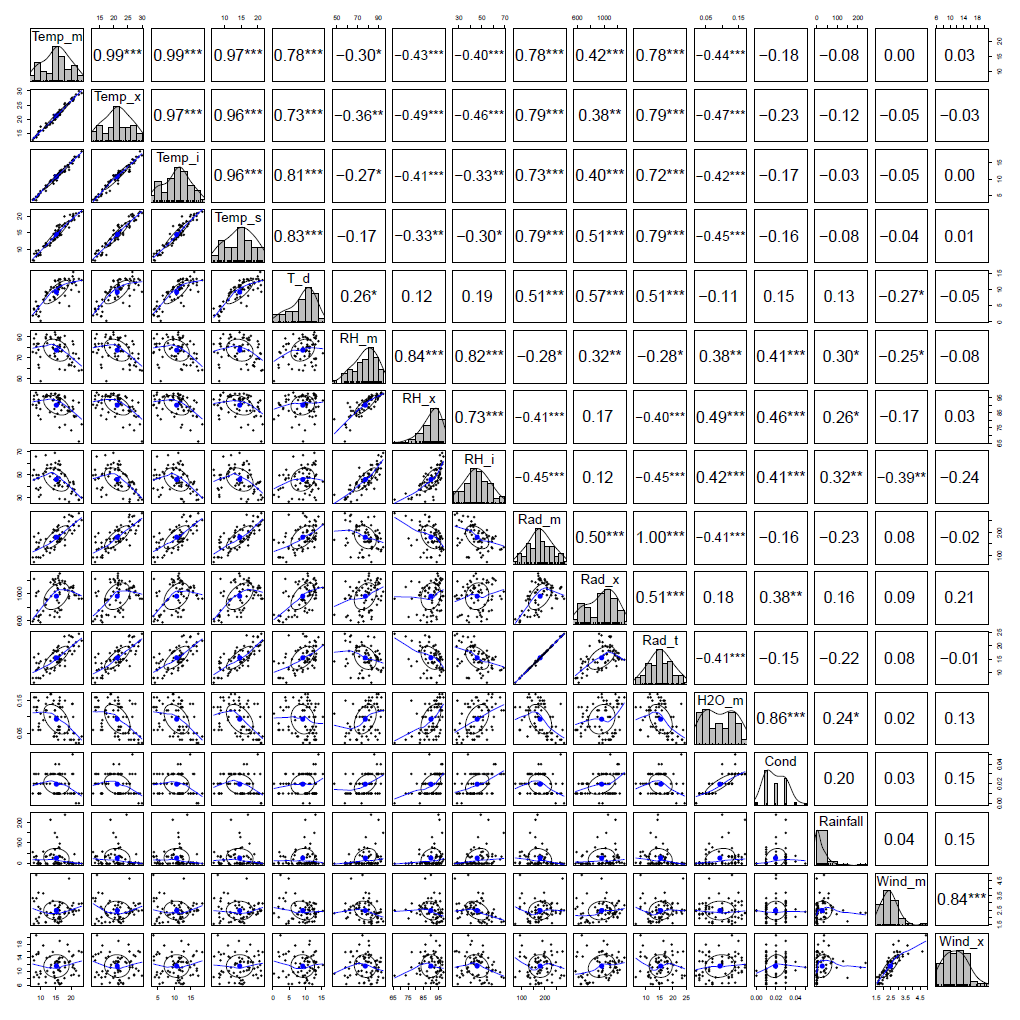
**FIGURE S3**. Pearson correlation matrix for climate variables considered in our study that potentially may influence SAVI spatial-temporal dynamics during the study period (from May to November during 2021-2023). Normality plots for each time series are also shown. The distribution of each variable is shown on the diagonal. Bivariate scatter plots with a fitted line are shown on the bottom of the diagonal. Symbols (“***”, “**”, “*”, “.”) show statistically significant correlations and the magnitude of the correlation (p-values 0.05, 0.01, 0.001, 0 respectively). To be more conservative, we set a threshold value of 0.75 above which we considered that two variables were highly correlated, and we retained only one of the two (in all cases, it was the mean value of the variable). Variable labels are abbreviated as follows: _m = mean, _x = maximum, _i = minimum; Rad_t = total radiation; Temp_s = mean soil temperature; Cond = soil conductivity; Rainfall = accumulated rainfall.


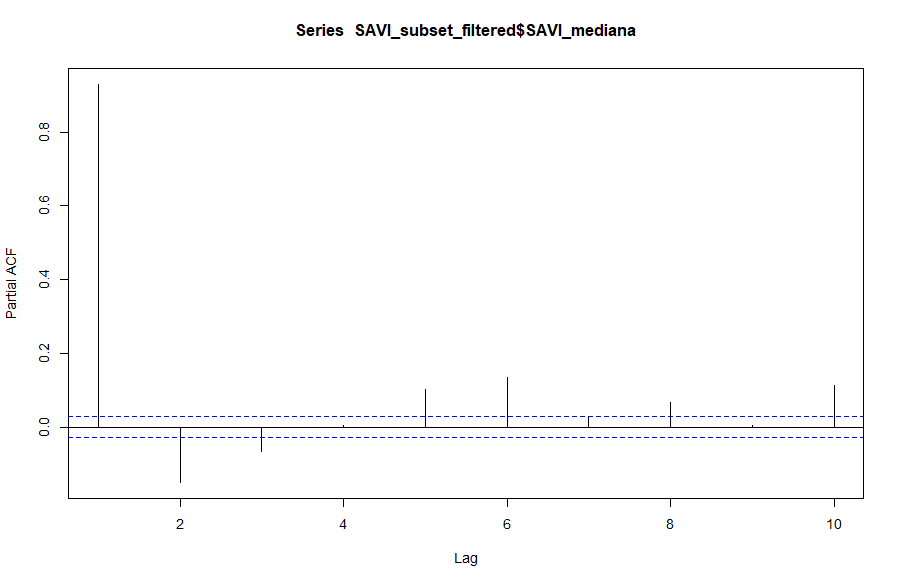


**FIGURE S4**. Results of the PACF analysis to assess at which temporal autocorrelation (shown as lag based on previous SAVI image values; 1 lag = 10 days) faded statistically. Results are shown for all 48 SAVI composites with 100 pixels randomly selected for each image. The dashed blue line shows the statistical significance threshold of 0.028, derived from the formula 1.96/sqrt(n) where n = 4800.

**
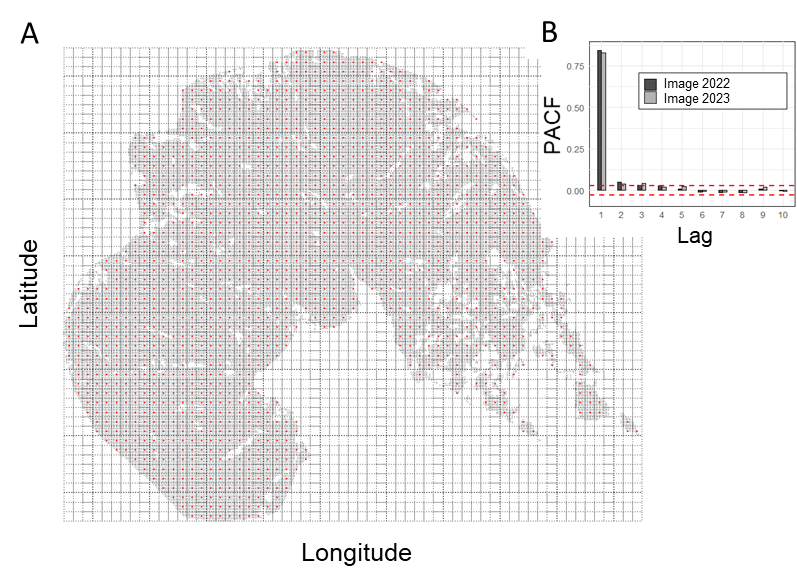
FIGURE S5**. (A) Results of the PACF analysis to assess at which spatial autocorrelation (shown as lag based on pixel windows) faded statistically. Results are shown for both SAVI images selected randomly, with 5000 pixels randomly selected for each image. The dashed red line shows the statistical significance threshold of 0.0277, derived from the formula 1.96/sqrt(n) where n = 5000. (B) Random selection of pixels considering that pixels cannot be at a lower distance than the one at which spatial autocorrelation was statistically significant, with this distance being 60m, and therefore taking the central pixel (red dots) of a 60x60m grid of spatial resolution (black grid). The cloud of grey points in the background corresponds to the centroids of the original 10m resolution pixels.


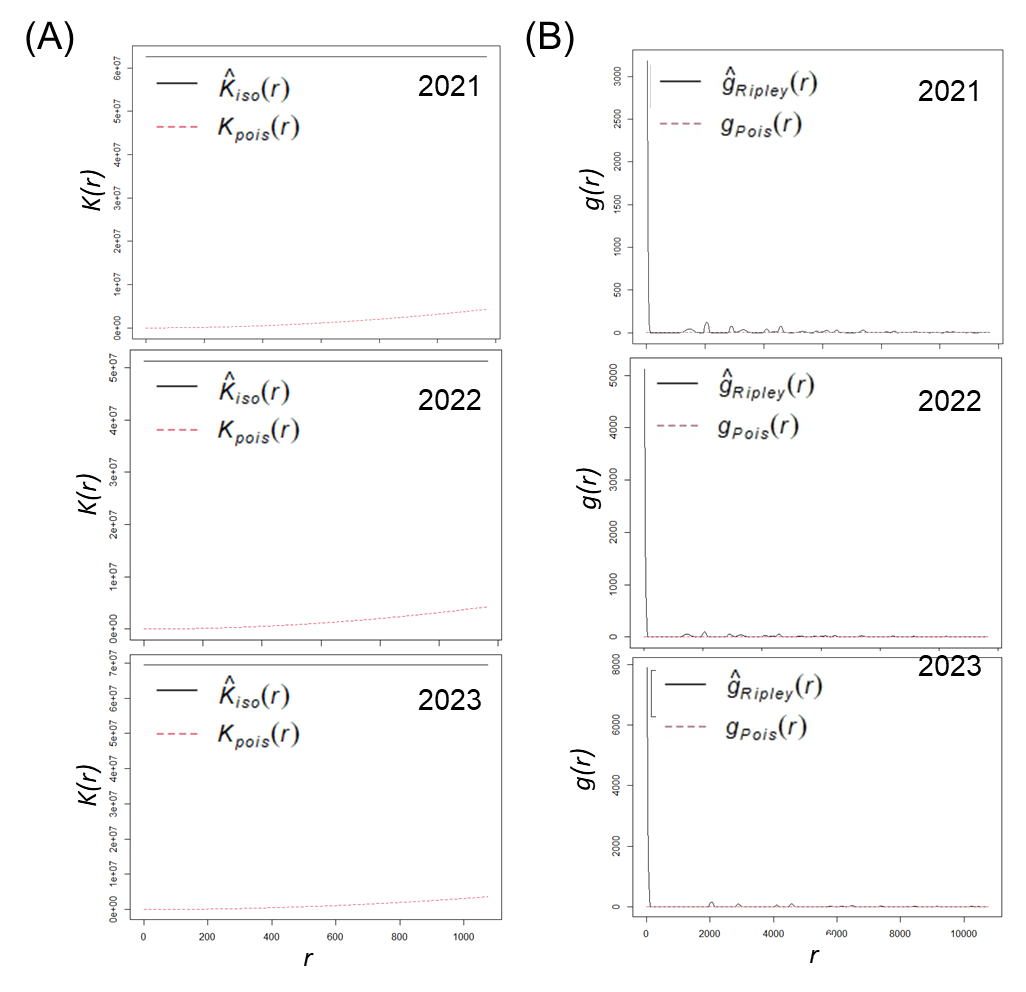


**FIGURE S6**. (A) Ripley’s K function plot to assess visually whether cows showed a clumped spatial pattern for the three years of data. The x-axis shows the parameter *r*, i.e. the spatial scale (distance in meters) at which the clustering was assessed, while *K(r)* is Ripley’s K function. The dashed red lines (*K_pois_(r)*) show the expected K function under a Poisson (CSR) process, i.e. a purely random spatial distribution. The solid black lines ($\hat{K}$*_iso_*(*r*)) are the observed *K* function from our dataset, i.e. the actual cow distribution. If cows were completely randomly distributed, the black curves would match the CSR lines. (B) Pair Correlation Function (PCF) *g(r)* for the three years of data: the black solid lines represent the observed *g*(*r*), while the red dashed lines show the expectation under a complete spatial randomness (CSR) model (i.e., a Poisson process). These panels show that cows tend to aggregate within small spatial scales, whereas at larger scales, spatial distributions are closer to random, i.e. at these large scales, cows do not show a clumped pattern.

| **Interval (dd-mm-yyyy)** | **Year** |
| --- | --- |
| 01-07-2021 to 10-07-2021 | 2021 |
| 01-07-2022 to 10-07-2022 | 2022 |
| 01-07-2023 to 10-07-2023 | 2023 |
| 09-09-2021 to 18-09-2021 | 2021 |
| 09-09-2022 to 18-09-2022 | 2022 |
| 09-09-2023 to 18-09-2023 | 2023 |
| 10-08-2021 to 19-08-2021 | 2021 |
| 10-08-2022 to 19-08-2022 | 2022 |
| 10-08-2023 to 19-08-2023 | 2023 |
| 11-06-2021 to 20-06-2021 | 2021 |
| 11-06-2022 to 20-06-2022 | 2022 |
| 11-06-2023 to 20-06-2023 | 2023 |
| 11-07-2021 to 20-07-2021 | 2021 |
| 11-07-2022 to 20-07-2022 | 2022 |
| 11-07-2023 to 20-07-2023 | 2023 |
| 19-07-2021 to 28-07-2021 | 2021 |
| 19-07-2022 to 28-07-2022 | 2022 |
| 19-07-2023 to 28-07-2023 | 2023 |
| 20-06-2021 to 29-06-2021 | 2021 |
| 20-06-2022 to 29-06-2022 | 2022 |
| 20-06-2023 to 29-06-2023 | 2023 |
| 21-08-2021 to 30-08-2021 | 2021 |
| 21-08-2022 to 30-08-2022 | 2022 |
| 21-08-2023 to 30-08-2023 | 2023 |
| 21-06-2021 to 30-06-2021 | 2021 |
| 21-06-2022 to 30-06-2022 | 2022 |
| 21-06-2023 to 30-06-2023 | 2023 |
| 29-06-2021 to 08-07-2021 | 2021 |
| 29-06-2022 to 08-07-2022 | 2022 |
| 29-06-2023 to 08-07-2023 | 2023 |
| 29-09-2021 to 08-10-2021 | 2021 |
| 29-09-2022 to 08-10-2022 | 2022 |
| 29-09-2023 to 08-10-2023 | 2023 |
| 30-08-2021 to 08-09-2021 | 2021 |
| 30-08-2022 to 08-09-2022 | 2022 |
| 30-08-2023 to 08-09-2023 | 2023 |
| 31-07-2021 to 09-08-2021 | 2021 |
| 31-07-2022 to 09-08-2022 | 2022 |
| 31-07-2023 to 09-08-2023 | 2023 |

**TABLE S1.** List of the 10‑day Sentinel‑2 SAVI median composites used in the study (2021–2023). Each interval represents a non‑overlapping 10‑day period for which cloud‑masked, gap‑filled median reflectance was computed.

| **QSSD** | **Frequency** | **Percentage** |
| --- | --- | --- |
| 0.0000 | 16616 | 51.19 |
| 223.9840 | 15810 | 48.71 |
| 316.7612 | 19 | 0.06 |
| 387.9517 | 11 | 0.03 |
| 619.7877 | 3 | 0.01 |
| 939.3588 | 1 | 0.003 |

**TABLE S2**. Frequency of positions and percentage for each QSSD value obtained from static (fixed to a pole) GPS device data. We defined the thresholds as follows: “Resting”: QSSD < 388, “No Class”: 388 ≤ QSSD ≤9 40, and “Grazing”: QSSD > 940.

|  | Climate | | | | | | Cows | |
| --- | --- | --- | --- | --- | --- | --- | --- | --- |
| Temporal window | Temp | RH | H2O | Rad | Rain | Wind | Grazing | Resting |
| 10 days | 47397 | 46833 | 43971 | 44718 | 45954 | 47379 | 46573 | 47198 |
| 20 days | 47293 | 47158 | 45343 | 45212 | 44625 | 47414 | 46740 | 47326 |
| 30 days | 46869 | 47250 | 46696 | 45682 | 44261 | 47229 | 46937 | 47388 |
| 40 days | 46199 | 47308 | 47209 | 46321 | 44514 | 47035 | - | - |
| 50 days | 45573 | 47370 | 47314 | 46782 | 46244 | 46451 | - | - |
| 60 days | 45415 | 47403 | 47310 | 46861 | 47344 | 46029 | - | - |

**TABLE S3**. AIC values of the models testing at which temporal window each variable better explained the variability in SAVI. All variables were standardized. Temp = temperature, RH = relative humidity, H2O = H_2_O soil content, Rad = solar radiation, Rain = cumulative precipitation, Wind = wind speed. Best models are highlighted in green.

| **COV** | **CURV** | **ORI** | **ELE** | **SLO** | **HYD** | **H2O** | **RAIN** | **RAD** | **RH** | **TEMP** | **WIND** | **REST** | **GRAZ** | **Dev** | **np** | **AIC** | Δ**AIC** | **weight** |
| --- | --- | --- | --- | --- | --- | --- | --- | --- | --- | --- | --- | --- | --- | --- | --- | --- | --- | --- |
| **+** |  | **+** | **+** | **+** | **+** | **+** | **+** | **+** |  | **+** | **+** | **+** | **+** | **-19146** | **23** | **38339** | **0** | **0.75** |
| + |  |  | + | + | + | + | + | + |  | + | + | + | + | -19151 | 20 | 38341 | 2.3 | 0.24 |
| + |  | + | + | + | + | + | + | + | + | + | + | + | + | -19150 | 24 | 38348 | 9.5 | 0.01 |
| + |  |  | + | + | + | + | + | + | + | + | + | + | + | -19154 | 21 | 38351 | 11.7 | 0.00 |
| + | + | + | + | + | + | + | + | + |  | + | + | + | + | -19151 | 24 | 38351 | 11.9 | 0.00 |
| + |  | + | + | + | + |  | + | + |  | + | + | + | + | -19154 | 22 | 38353 | 14.0 | 0.00 |
| + | + |  | + | + | + | + | + | + |  | + | + | + | + | -19156 | 21 | 38353 | 14.2 | 0.00 |
| + |  |  | + | + | + |  | + | + |  | + | + | + | + | -19159 | 19 | 38355 | 16.4 | 0.00 |
| + | + | + | + | + | + | + | + | + | + | + | + | + | + | -19155 | 25 | 38360 | 21.3 | 0.00 |
| + | + |  | + | + | + | + | + | + | + | + | + | + | + | -19159 | 22 | 38363 | 23.7 | 0.00 |

**TABLE S4**. List of the 10 best models after testing for all 16384 possible additive combinations of the 24 explanatory variables (results not shown). Variables and their abbreviations are listed by groups (see Methods for details): vegetation types (COV, highlighted in orange); topographic variables (highlighted in yellow): curvature (CURV), orientation (ORI), elevation (ELE), slope (SLO), and hydrology (HYD); climate variables (highlighted in blue): H2O soil content (H2O), cumulative precipitation (RAIN), radiance (RAD), relative humidity (RH), temperature (TEMP), and wind velocity (WIND); and cow activity (highlighted in green): cow resting (REST) and grazing (GRAZ). For dynamic variables (climate and cow activity), the variables in the models here were those selected for temporal inertial windows. Dev = Deviance of the model; np = number of parameters; AIC = Akaike Information Criterion; ΔAIC = difference of AIC value relative to the best model; weight = weight of each model to total weight (1). A + symbol means that the variable was included in the model, whereas grey cells show that the parameter was not included in the model. Models are ranked by their AIC value, and the best selected model is in bold.

| Model | Deviance | np | AIC | ΔAIC |
| --- | --- | --- | --- | --- |
| **Grazing * Orientation** | **38040** | **25** | **38322** | **0** |
| **Grazing * Elevation** | **38065** | **23** | **38335** | **12,8** |
| **Grazing * Slope** | **38071** | **23** | **38341** | **19,4** |
| *Full model* | *38087* | *22* | *38343* | *21,5* |
| Resting * Elevation | 38077 | 23 | 38346 | 24,3 |
| Resting * Slope | 38082 | 23 | 38351 | 29,6 |
| Grazing* Hydrology | 38083 | 23 | 38352 | 30,5 |
| Resting * Hydrology | 38087 | 23 | 38356 | 34,5 |
| Resting * Orientation | 38075 | 25 | 38358 | 36,0 |
| Grazing * Vegetation type | 38029 | 29 | 38361 | 39,4 |
| Resting * Vegetation type | 38066 | 29 | 38393 | 70,8 |

**TABLE S5**. Modelling interactions between grazing and the variables belonging to the static categories (topography and vegetation type). Models performing better than the full model (in italics) are in bold. Np = number of parameters; AIC = Akaike Information Criterion; ΔAIC = difference of AIC value relative to the best model
